# Investigation of Molecular Features Involved in Clinical Responses and Survival in Advanced Endometrial Carcinoma Treated by Hormone Therapy

**DOI:** 10.3390/jpm12050655

**Published:** 2022-04-19

**Authors:** Mathias Neron, Arnaud Guille, Lucie Allegre, Pierre-Emmanuel Colombo, Cristina Leaha, José Adelaide, Nadine Carbuccia, Frédéric Courtier, Florence Boissiere, Evelyne Crapez, Michel Fabbro, Sébastien Gouy, Emilie Mamessier, Éric Lambaudie, Daniel Birnbaum, François Bertucci, Max Chaffanet

**Affiliations:** 1 Department of Surgical Oncology, Institut du Cancer de Montpellier, Univ Montpellier, 34000 Montpellier, France; mathias.neron@icm.unicancer.fr (M.N.); pierre-emmanuel.colombo@icm.unicancer.fr (P.-E.C.); 2 Montpellier Cancer Research Institute (IRCM), Univ Montpellier, Inserm, Montpellier Cancer Institute (ICM), 34298 Montpellier, France; 3 Predictive Oncology Laboratory, Marseille Cancer Research Centre (CRCM), Inserm U1068, CNRS UMR7258, Institut Paoli-Calmettes, Aix-Marseille University, Label “Ligue Contre le Cancer”, 13009 Marseille, France; guillea@ipc.unicancer.fr (A.G.); adelaidej@ipc.unicancer.fr (J.A.); nadine.carbuccia@inserm.fr (N.C.); frederic.courtier@crchudequebec.ulaval.ca (F.C.); emilie.mamessier@inserm.fr (E.M.); lambaudiee@ipc.unicancer.fr (É.L.); daniel.birnbaum@inserm.fr (D.B.); bertuccif@ipc.unicancer.fr (F.B.); 4 Department of Obstetrics & Gynecology, Nimes University Hospital, 30029 Nimes, France; lucie.allegre@chu-nimes.fr; 5 Department of Pathology, Institut du Cancer de Montpellier, 34000 Montpellier, France; cristina.leaha@icm.unicancer.fr; 6 Translational Research Unit, Institut du Cancer de Montpellier, 34000 Montpellier, France; florence.boissiere@icm.unicancer.fr (F.B.); evelyne.crapez@icm.unicancer.fr (E.C.); 7 Department of Clinical Oncology, Institut du Cancer de Montpellier, 34000 Montpellier, France; michel.fabbro@icm.unicancer.fr; 8 Department of Surgical Oncology, Gustave Roussy Institute (IGR), 94800 Villejuif, France; sebastien.gouy@gustaveroussy.fr; 9 Department of Surgical Oncology, Institut Paoli-Calmettes, 13009 Marseille, France

**Keywords:** metastatic endometrial carcinoma, hormone therapy, predictive factors, mutations, copy number alterations, protein expression

## Abstract

Hormone therapy (HT) is an effective treatment for metastatic endometrial carcinoma (mEC), with limited toxicity and low cost. We focused on molecular analysis of mECs treated by HT and, for the first time to date, we compared the genomic profiles of paired metastasis and primary ECs. The main objective was to identify predictive factors of the response to HT as well as specific altered signaling pathways driving mEC biology. From 1052 patients with EC treated by HT in two French cancer centers, 32 with endometrioid EC and 6 with high grade serous EC were included. We evaluated hormone receptors (HR) and mismatch repair proteins expression by immunohistochemistry and gene alterations by targeted next-generation sequencing and array-based comparative genomic hybridization. Several variables were tested in univariate and multivariate analyses to identify potential associations with (i) the clinical benefit of HT (CBHT) and (ii) a longer response (>18 months) (LRHT) and overall survival (OS). We compared the biological and genomic profiles of 11 primary/metastatic EC pairs. Thirty tumors (78.9%) were HR-positive and 6 (15.8%) showed microsatellite instability (MSI). The genomic profiles of 34 tumors showed an average altered genome of 3.26%, DNA repair homologous recombination deficiency in five tumors (14.7%), and 17 regions significantly targeted by amplification/deletion. Thirty-three tumors had 273 variants (158 genes, median of 7 mutations/sample), including 112 driver mutations. *TP53*, *PTEN*, *PPP2R1A*, *ARID1A*, *FGFR2*, and *PIK3CA* were the most frequently mutated. Based on the genomic status, nine oncogenic pathways were altered in more than 25% of primary EC. Clinically, 22 (57.9%) and 6 (15.8%) patients presented CBHT and LRHT, respectively. Neither oncogenic pathways alterations nor the variables tested were associated with CBHT and LRHT. Only patient’s age, mitotic index and the presence of at least one HR were associated with OS. Paired analysis of the primary/metastatic samples showed that among the 22 mutations acquired in the metastatic counterparts, the most frequently targeted genes were involved in pathways that might confer a selective advantage to cancer metastasis including hormone resistance. In conclusion, only patient’s age, mitotic index and the presence of at least one HR were associated with OS. The identification of gene mutations newly acquired in metastasis might help to better understand the formation of EC metastasis and select the best actionable candidates for HT-treated patients at the metastatic stage.

## 1. Introduction

In 2020, endometrial carcinoma (EC) was the sixth most common malignancy in women and the second most common gynecological cancer after cervical cancer, with an incidence of 11.1 cases for 100,000 women and a mortality rate of 2.1 for 100,000 women [1]. ECs are stratified according to the World Health Organization (WHO) classification system into several pathological subgroups including endometrioid, serous, clear cell, mixed cell adenocarcinoma, and other relatively rare types such as mucinous adenocarcinoma, neuroendocrine tumors, dedifferentiated carcinoma, and undifferentiated carcinoma. Although overall prognosis is good, EC recurrence is observed in about 20% of patients [2].

The management of metastatic EC (mEC) is not well codified and may include chemotherapy and hormone therapy (HT). HT should be considered as a first-line treatment because of its low toxicity in a population of patients with frequent co-morbidities (e.g., obesity/overweight, diabetes) [3]. The response rate to progestins (medroxyprogesterone acetate mainly) in mEC is about 25%, but the clinical benefit rate can reach 56% [4,5]. To date, there is no predictive factor of the response to HT; the assessment of the hormone receptor (HR) status is not necessary [6,7,8] and has been rarely assessed in the literature [9]. Nevertheless, it has been suggested that progesterone receptor (PR) expression in the tumor could be a predictive factor of the response to HT and should be tested before treatment initiation [3]. However, to optimize the identification of patients who will benefit from HT, additional predictive biomarkers of HT response are needed [8].

As illustrated by some studies in ovarian and other cancers [10,11,12], high-throughput molecular analyses now allow the rapid and effective evaluation of the tumor profile to tailor adjuvant therapy and to predict the response to treatments. Concerning EC, molecular classification, mainly based on whole-exome sequencing, was derived from associations with pathological features [13], before being recently simplified to be clinically applicable [14,15] and suggesting the use of emerging targeted therapies [16]. However, to date, no molecular stratification of EC is currently used in the routine to adapt adjuvant therapy or to predict the therapeutic response. The objective of this retrospective study was to identify predictive factors of the response to HT in mEC by applying molecular and immunohistochemistry (IHC) analyses. We also compared the molecular profiles of 11 paired primary tumor/metastatic samples.

## 2. Materials and Methods

### 2.1. Patients

This retrospective study involving two French comprehensive Cancer Centers, the Institut du Cancer de Montpellier (Montpellier, France) and the Institut Paoli-Calmettes (Marseille, France), concerned patients treated between January 2000 and January 2016. The medical files of all patients with mEC treated during this period were reviewed. Inclusion criteria were: mEC, endometrioid or serous type, treated by HT, and availability of formalin-fixed paraffin-embedded (FFPE) primary and metastatic tumor samples. Exclusion criteria were: HT for an associated breast cancer, and HT response not evaluable. The study was approved by the local translational research committee (CORT-27062016). Patients gave their signed informed consent.

### 2.2. Tumor Samples

FFPE samples of the primary tumor, and of the metastatic tumor if possible, were analyzed. First, the local expert in gynecologic pathology confirmed the EC pathological type and selected the tumor areas with at least 30% of cancer cells for genomic analysis after examination of hematoxylin and eosin-stained tumor sections. Then, estrogen receptor (ER), progesterone receptor (PR), and microsatellite instability (MSI) status (based on the expression of the mismatch repair proteins MLH1, MSH2, MSH6, and PMS2) were assessed by IHC using antibodies and protocols described in Table S1. MSI was also determined with the fluorescent multiplex PCR-based Microsatellite Instability Analysis system, v. 1.2 (Promega) with DNA extracted from the FFPE blocks. For all tumors, MSI status was assessed by both IHC and PCR testing, as usually performed in our centers for EC due to the possible lack of sensitivity of IHC [17]. Loss of expression was recorded when the nuclear staining was absent in all cancer cells and preserved in normal epithelial and stromal cells. A tumor was considered as ER and/or PR positive when ≥10% of tumor cells were positive. Mitotic count corresponded to the number of mitotic figures observed in 10 high-power fields (×400). For molecular analyses, tumoral DNA was extracted from FFPE tumor samples (10 sections of 5 µm per sample; mean percentage of cancer cells = 60%) using the QIAamp DNA FFPE Tissue Kit from QIAGEN (QIAamp DNA Stool Mini Kit, QIAGEN, Courtaboeuf, France), according to the manufacturer’s instructions. Quality was verified on polyacrylamide gel electrophoresis, and concentration assessed by using Qubit dsDNA BR Assay.

### 2.3. Targeted Next-Generation Sequencing (tNGS)

Mutation profiles were established by using tNGS as previously described [18]. For each sample, a library of all coding exons and intron–exon boundaries of 495 cancer-associated genes (custom panel, “TSV9 FFPE”, Agilent Design ID: 27066-1418742171 described in Table S2) was constructed using the HaloPlex Target Enrichment System (Agilent Technologies, Santa Clara, CA, USA) as previously described [18]. The custom-made panel was built with 495 genes selected for their involvement in cancers including the most frequently mutated genes in endometrial carcinomas (i.e., *ARID1A, ARID5B, CTNNB1, KRAS, PIK3CA, POLE, PTEN*, and *TP53*) [13,19] as well as *ESR1* and DNA repair genes (MMR, homozygous recombination, etc.). Its design was optimized for FFPE samples with help of Agilent Technologies. Libraries were qualified and quantified on Bioanalyzer (Agilent Technologies, Massy, France) and QuBit fluorometer (Thermo Fischer Scientific, Breda, Netherlands) before being sequenced (2 × 150 bp paired ends reads) using the Illumina MiSeq platform according to the manufacturer’s instructions (Illumina, San Diego, CA, USA), as previously described [18].

The sequence data were aligned to the human reference genome (UCSC hg19) using Burrows–Wheeler Aligner [20]. Tumor samples were sequenced at an average depth of 640× (range, 23 to 1853) for the targeted regions. Bam files were processed as described [18]. Single nucleotide variants (SNVs) calling was performed with Varscan2 version v2.3.8 [21] with a minimal alternate variant frequency and coverage set at 0.02 and 10, respectively. Insertions/deletions (indels) calling was performed using GATK haplotypecaller version 3.7 [22] with default parameters and pindel [23]. The variants, i.e., SNVs and indels, were annotated with the Annotate Variation Software (ANNOVAR, version 2013-11-12). Known variants found in dbsnp129 and dbsnp137 with a variant allele frequency (VAF) superior to 1% (1000 G or ESP6500) were removed. Finally, low frequency SNVs and indels that were suspected to be false positives were systematically inspected with IGV version 2.3.32 [24,25]. Mutations were classified as “neutral” or “damaging” using the majority rule of predictor software (provided by dbnsfp: Sift, Polyphen2, LRT, MutationTaster, MutationAssesor, FATHMM, RadialSVM, LR) as previously described [26]. A “recurrent” mutation, also called “hot spot”, was defined as being found more than 10 times in the Catalogue of Somatic Mutations in Cancer (COSMIC V68) database (http://cancer.sanger.ac.uk/cosmic, accessed on 30 May 2017); the other variants were defined as “non-recurrent”. Driver mutations were established by using Cancer Genome Interpreter (CGI, https://www.cancergenomeinterpreter.org/home, accessed on 6 May 2021).

### 2.4. Array Comparative Genomic Hybridization (aCGH)

The genomic profiles were established by using aCGH onto high-resolution 4 × 180 K CGH microarrays (SurePrint G3 Human CGH Microarray Kit, Agilent Technologies, Massy, France). For each patient, tumor DNA was co-hybridized with a pool of human normal female DNAs used as reference (G1471 Promega). Microarrays were scanned with an Agilent Autofocus Dynamic Scanner (G2565BA, Agilent Technologies, Massy, France). Data were analyzed and visualized with the CGH Analytics 3.4 software (Agilent Technologies, Massy, France) as previously described [18,27]. All probes for aCGH were mapped according to the hg19/NCBI human genome mapping database. For each gene, the DNA copy number alteration was established using two different threshold values (log2 ratio > |0.5| and |1|) to distinguish low (gain/loss) from high (amplification/deletion) level copy number alterations (CNA), respectively [27]. 

The percentage of genome altered was calculated as the sum of altered probes divided by the total number of probes after removing sexual chromosomes. GISTIC analysis was performed with alteration threshold set to 1 for both amplifications and deletions. Copy number profiles of EC samples were categorized into copy number-low and copy number-high according to the mean value of percentage of genome altered as previously described [13]. Then, to simplify the statistical analysis due to the low number of included samples, data were grouped in two categories: gain/amplification and loss/deletion. For each tumor, a homologous recombination deficiency (HRD) score (HRD aCGH score), based on losses of heterozygosity (LOH), was calculated [28]. A score ≥10 was considered as HRD-high.

In addition to the analysis of individual genes, we also analyzed 20 oncogenic pathways. These latter are defined in Table S3: DNA damage repair pathway (BER, NER, MMR, FA, HRD, NHEJ, DR, TLS, NP, OTHER) and pathways related to Cell Cycle, HIPPO, MYC, NOTCH, NRF2, PI3K, RTK_RAS, TGFβ, TP53, and WNT were assembled from data previously reported [29,30]. For each gene included in a pathway, we defined its role as oncogene, tumor suppressor gene (TSG), or undetermined. Based on gene mutation and CNA targeting oncogenic pathways, potential altered oncogenic pathways were identified. For an oncogene, the alteration was retained only when it was mutated or amplified. For a TSG, the alteration was retained only when it was mutated or lost/deleted. For an undetermined gene, the alteration was retained only when it was mutated, amplified, or lost/deleted. A pathway was considered altered when at least one gene in this pathway was altered.

### 2.5. Statistical Analysis

The primary clinical endpoint was the clinical benefit of HT (CBHT) in the metastatic setting, defined as a complete or partial response to HT or stable disease for ≥6 months from the start of HT, according to the RECIST 1.1 criteria. This endpoint was used in previous studies on HT in mEC [5]. Secondary clinical endpoint was long-lasting response to HT (LRHT), defined as complete or partial response for ≥18 months from the start of HT. Overall survival (OS) was defined as the time from the first metastatic relapse to death from any cause.

The patients’ characteristics are described using frequencies (%, *n*) for qualitative data, and medians (interquartile range) for quantitative data. Continuous variables were compared using the Wilcoxon or Kruskal—Wallis test. Categorical data were compared using the Fisher’s exact test. The median follow-up was calculated with the reverse Kaplan–Meier method from the start of metastatic event. Survival rates were estimated with the Kaplan–Meier method [31], and presented with their 95% confidence intervals (95% CI). Univariate and multivariate prognostic analyses were conducted using the Cox regression model. All variables with *p* < 0.1 in univariate analysis were included in the multivariate analysis. Variables including clinical and biological features as well as genes mutated in at least 3 samples were tested for association with CBHT in univariate and multivariate analyses by using the logistic regression model. All statistical analyses were conducted with R 3.5.1 (R Development Core Team (2018) R Foundation for Statistical Computing, Vienna, Austria). The level of significance was set at *p* < 0.05 and all tests were two-sided.

## 3. Results

### 3.1. Patients and Samples Characteristics

A total of 38 patients with mEC and available primary tumor samples were retrospectively selected. Paired samples of both the primary and metastatic tumors were available for 11 patients. The patients’ characteristics are summarized in Table 1. The median overall survival (OS) was 40 months (95% CI: 14–74) (Figure S1). The median follow-up was 155 months from the date of the first metastatic relapse. Twenty-one patients (54%) received HT as a first-line treatment for the metastatic disease (without any previous chemotherapy). Progestins were the main HT (71.1%). Other patients received HT in the metastatic setting in the 2nd, 3rd, 4th, or 5th line. All EC samples were analyzed by our local expert pathologist in gynecologic oncology. This analysis confirmed that 32 patients (84.2%) had endometrioid EC and 6 (15.8%) high grade serous EC (HGSEC). IHC analysis showed the positivity of at least one HR (ER or PR) in 30/38 (78.9%) samples. Moreover, six tumors (15.8%) displayed an MSI status.

### 3.2. Genomic Alterations of Tumor Samples

**Figure 1:**
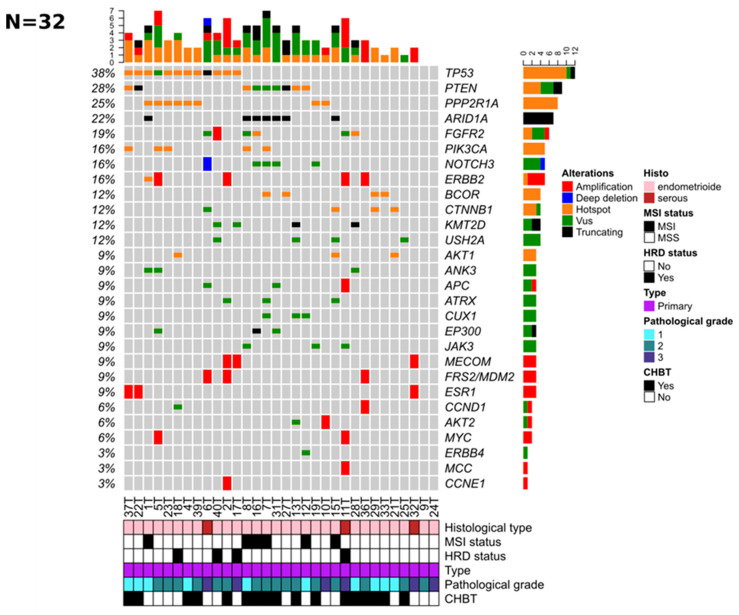
Genomic alterations mainly target oncogenic driver genes in 32 primary ECs. Oncoprints of the 28 frequently altered genes were analyzed in 32 samples: Somatic alterations (mutations or high CNAs (amplification, deep deletion)) are color-coded according to the legend (right part). The genes (rows) are ordered from top to bottom by the decreasing number of altered tumors (right panel), and tumors (columns) are reordered to visualize the mutual exclusivity between samples. The total number of molecular alterations is indicated for each sample (top panel). Among them, a somatic hotspot mutation is defined as a variant with at least 10 occurrences in the COSMIC database [https://cancer.sanger.ac.uk/cosmic, accessed on 30 May 2017] and VUS is a variant of unknown significance. Unique and anonymized patient numbers are indicated. For each sample, the histological type, MSI status, HRD status, pathological grade, and CHBT status are indicated at the bottom of the figure (see corresponding legend on the right part).

Among the 38 selected patients, DNA extraction from primary tumor samples failed for three patients and NGS data of two samples did not meet the quality criteria and were not interpretable due to low coverage. Thus, aCGH and tNGS data were available for 34 and 33 patients, respectively. Both aCGH and tNGS data were available in 32 patients (Figure 1). After filtering, we retained 273 variants (248 SNVs and 28 indels) involving 158 genes (Table S4). Among them, 112 variants (41%) were annotated as driver mutations (TIER1 and TIER2) by the cancer genome interpreter (CGI). The mean number of mutations per sample was seven. The six most frequently mutated genes were *TP53* (36%), *PTEN* (30%), *PPP2R1A* (24%), *ARID1A* (21%), *FGFR2* (18%), and *PIK3CA* (15%). Two *POLE* variants of unknown significance (VUS) were identified in two samples (see Table S4). The mean percentage of altered genomes observed by aCGH in the 34 tumors was 3.26%. GISTIC analysis identified 16 regions of amplifications and one region of deletion significantly altered (FDR < 0.05) (Tables S5 and S6, respectively). Among them, four amplified genes are known to act as drivers by CGI: *ESR1* (6q25.1), *MDM2* (12q15), *ERBB2* (17q12), and *AKT2* (19q13.2) (Table S7). The HRD profile was observed in five tumors (14.7%).

We then searched for potential correlations between genomic profiles (CNA), pathological type, mutation, and MSI status. HGSEC tended to present a higher percentage of altered genomes than endometrioid EC (*p* = 0.103, Wilcoxon test). Profiles with *TP53* mutation were associated with a higher percentage of altered genomes (*p* = 0.005, Wilcoxon test) than those without *TP53* mutation. Conversely, profiles with *PTEN* mutation were associated with a lower percentage of altered genomes (*p* = 0.026, Wilcoxon test) than those without *PTEN* mutation. Patients with MSI profile were associated with a higher number of gene mutations (*p* = 0.008, Wilcoxon test). *TP53* and *PTEN* mutations were significantly observed in high and low CNA profiles, respectively (*p* = 0.006 and *p* = 0.034, Fisher test, respectively). This suggests that *TP53* and *PTEN* mutations might be involved in distinct tumorigenic mechanisms. Based on gene mutations and CNAs, RTK_RAS (75%), OTHER DNA damage repair (66%), PI3K (62.5%), NOTCH and TP53 (44%), WNT and HRD (31%), FA (28%), and HIPPO (25%) oncogenic pathways were identified as being altered in more than 25% of primary EC, whereas the Cell Cycle, BER, NER, and MYC pathways were altered in more than 10% of cases (Table S8).

### 3.3. Clinical Benefit to HT (CBHT)

CBHT was observed in 22 out of 38 patients (58%). The median duration of CBHT was 6 months (range: 3–10). To identify variables associated with CBHT, we tested different variables including clinical and molecular features: the genes mutated and oncogenic pathways altered in at least three samples. Results from univariate and multivariate analyses are shown in Table 2. In univariate analysis, variables including body mass index >30 (OR = 1.18; *p* = 0.04), mitotic count (OR = 0.95; *p* = 0.04), at least one positive HR (OR = 6; *p* = 0.05), and percentage of altered genome (OR = 0.61; *p* = 0.03) were found to be associated with CBHT, while *PTEN* mutation as well as NOTCH and HIPPO altered oncogenic pathways showing a trend (*p* < 0.1). In multivariate analysis, none of these variables remained significantly associated with CBHT.

### 3.4. Durable Clinical Response to HT (LRHT) and Overall Survival

Six patients (15.8%) presented LRHT. To identify variables associated with LRHT, we tested variables including clinical and molecular features as well as genes mutated and oncogenic pathways altered in at least three samples. The results from univariate analyses are shown in Table 3. In univariate analysis, only *PTEN* mutations were found to be associated with LRHT (OR = 7; *p* = 0.048). The multivariate analysis was irrelevant and non-informative because *PTEN* mutation status was the only significant variable. 

With a median follow-up of 155 months, 32 patients (84%) died. The median overall survival (OS) was 40 months (95% CI: 14–74). To identify variables associated with OS, we tested variables including clinical and molecular features as well as genes mutated and oncogenic pathways altered in at least three samples. The prognostic factors for OS retained after univariate analysis (Table 4) were age (HR = 1.10; *p* = 0.002), mitotic index (HR = 1; *p* = 0.006), PR expression status (HR = 0.38; *p* = 0.01), presence of at least one positive HR (HR = 0.29; *p* = 0.004), and percentage of altered genome (HR = 1.1; *p* = 0.029). In multivariate analysis, patient’s age (HR = 1.08; *p* = 0.008), mitotic index (HR = 1.04; *p* = 0.037), and the presence of at least one hormone receptor (HR = 0.21; *p* = 0.003) remained significant.

### 3.5. Comparison of Paired Primary and Metastatic EC Samples

**Figure 2:**
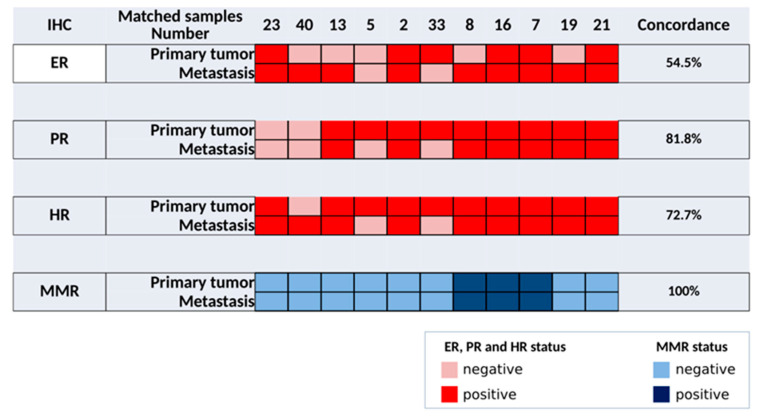
Matched primary and mEC show a high concordance level for HR and MMR. Hormone receptor expression was considered positive if ≥10% of cells were positive. Red: positive immunohistochemistry or amplification; dark blue: MSI. IHC: immunohistochemistry; ER, estrogen receptor; PR, progesterone receptor; HR: expression of at least one hormone receptor; MMR, mismatch repair proteins.

Eleven, seven, and eleven pairs of primary tumor and metastatic samples were profiled by IHC, aCGH, and tNGS, respectively. The results of IHC data are summarized in Figure 2. Only 54.5% of concordance for ER expression was observed, with 5/11 discordant pairs. From primary tumors to paired metastasis, one and four pairs changed from positive to negative and from negative to positive ER status, respectively. Eighty-two percent of concordance was observed for PR expression, with 2/11 discordant pairs. From primary tumors to paired metastasis, both changed from positive to negative PR status. In these two mEC samples, PR expression slightly decreased (from 10% to 5%) compared with the matched primary tumor, but this was sufficient to change the PR status (threshold 10%). Overall, 73% of concordance for HR expression was observed with 3/11 discordant pairs. From primary tumors to paired metastasis, two and one pairs changed from positive to negative and from negative to positive HR status, respectively. Concordance was 100% between the primary tumor and metastasis for the MMR status.

**Figure 3:**
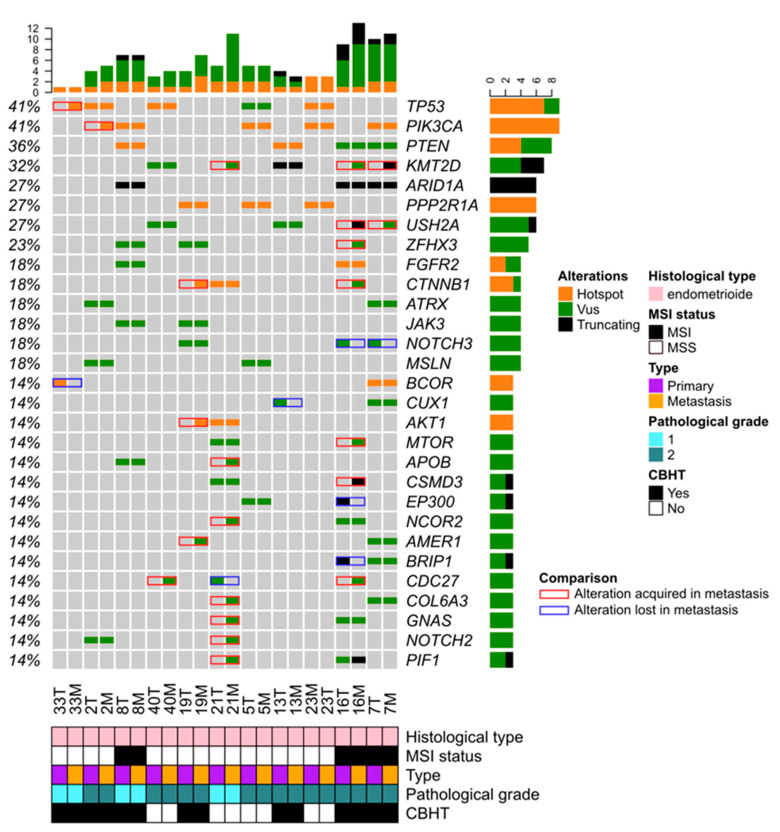
Matched primary and mEC show 22 and 7 mutations acquired and lost in metastatic counterparts, respectively. Legend: green, variant of unknown signification (VUS); orange, hotspot; black, truncating mutation; grey, wild. The right column highlights the difference between the matched samples. Changes over time are coded by: red, mutation gain; green, mutation loss; grey, result not available. Only the 29 most mutated genes are represented.

The genomic profiles of the seven pairs of primary and metastatic samples established by aCGH were compared (data not shown). Save for one *TP53* deletion observed in metastatic sample 16 M, no other relevant CNA was acquired in the metastatic counterparts. By contrast, the comparison of tNGS data showed 29 mutational changes in 8/11 pairs (Figure 3). MSI IHC status as well as *TP53*, *PIK3CA*, *PTEN*, *ARID1A*, *PPP2R1A*, *FGFR2*, *ATRX*, and *JAK3* mutations were mainly found maintained in the metastatic counterparts. In seven pairs (n° 2, 7, 16, 19, 20, 33, and 40), 22 mutations targeting 17 genes were acquired in the metastatic counterparts. Among them, somatic mutations affecting genes involved in PIK3/AKT/MTOR signaling (*PIK3CA*, *AKT1*, and *MTOR*), histone methyltransferase (*KMT2D*), the cell cycle regulator (*CDC27*), and WNT/β-catenin pathway (*CTNNB1*) were the most frequent, suggesting they might confer a selective advantage to cancer metastasis. In five pairs (n°7, 13, 16, 21, and 33), seven mutations targeting six genes (*BCOR*, *BRIP1*, *CDC27*, *CUX1*, *EP300*, and *NOTCH3*) were lost in the metastatic counterpart. This suggests that such mutations might have been included in another or others clones lost under treatment and that they were probably not drivers of the metastatic process. 

Based on gene mutations and CNAs, PI3K, RTK_RAS, OTHER DNA damage repair pathways, HIPPO, NOTCH, WNT, NER, HRD, MYC, TP53, BER, FA, and NHEJ oncogenic pathways were identified as being altered in more than 25% of metastatic samples (*n* = 7) (Table S8). In a first global comparison of the frequencies of altered oncogenic pathways with primary EC tumors (*n* = 32), the only difference was a trend towards more frequent PI3K pathway alteration in metastatic samples (100% versus 75%; *p* = 0.077; Table S8). A second comparison was performed only on the seven pairs of primary and metastatic samples (Table S9). Through the 14 samples, a pathway was altered 51 times. Of these, in 31 alterations, the pathway was altered in both primary tumors and paired metastases, and the PI3K pathway was the most frequent pathway altered in all 14 samples; in 19 times, the pathway was altered in the metastasis but not in the paired primary tumor, and the NER pathway was the most frequently diverging (3/7 pairs); in one alteration, a pathway was altered in the primary tumor but not in the paired metastasis. Overall, in 4/7 pairs, additional pathways appeared to be altered in the metastasis.

## 4. Discussion

In this retrospective study, clinical and biological variables were simultaneously assessed to identify predictive factors of the response to HT in patients with metastatic EC. The place of HT in EC is not consensual but represents an effective and safe oral therapy for these patients. In our study, 58% of patients reported a clinical benefit from HT, a rate similar to the 54.1% reported in a prospective trial studying megestrol acetate as a control in advanced EC. Thus, despite a high proportion of endometrioid ECs being ER and/or PR positive, endocrine therapy is only effective in a minority of women with EC, and, ultimately, patients progress with resistant disease. Several studies using endocrine therapy in EC have been reported [32,33,34,35]. While some potential biomarkers were suggested, none are currently used in routine clinical pratice. The genomic profiling of tumor samples might identify new potential therapeutic targets and contribute to optimizing endocrine therapy in EC. To our knowledge, our series is the first to assess the prognostic/predictive value of tNGS and aCGH in patients with EC treated with HT in the metastatic setting.

Genomic profiling. Genomic data established on our EC sample series were globally consistent with those observed in larger studies on EC biology [13,19,36,37,38]. We reported (i) *TP53*, *PTEN*, *PPP2R1A*, *ARID1A*, *FGFR2*, and *PIK3CA* as the most frequently mutated genes, (ii) 16 regions were significantly amplified, including *ESR1* (6q25.1), *MDM2* (12q15), *ERBB2* (17q12), and *AKT2* (19q13.2), all known as oncogene drivers, (iii) an HRD profile in five tumors (14.7%), and (iv) HGSEC with a tendency to present a higher percentage of altered genomes than endometrioid EC. We noted that the comparison with MSKCC genomic data showed some significant frequency differences among commonly altered genes (Tables S10 and S11) [36]. This could be explained by the proportions of histological types present in the two EC cohorts. Indeed, the highest frequency of *PPP2R1A* mutations (6 and 2 hot spot variants, P179R and R183W, respectively) observed in our study might suggest that our cohort is more enriched in serous histology in which *PPP2R1A* mutations are more common. Moreover, we cannot exclude that such results might also be explained in part by the ethnic diversity related to the pathogenesis of cancer [39].

Genomic alterations in primary EC might explain endocrine failures. Among gene alterations, *ESR1* amplification and other targeted genes involved in signaling pathways (PI3K/AKT/MTOR, ERBB2, FGFR, CDK4/CDK6, MDM2–TP53, histone deacetylases) are known to cross-talk with the ER pathway in breast cancer [40]. Their alteration in EC suggests that they are responsible for endocrine failure in both BC and EC cancers. Encoded by the *ESR1* gene, the ERα protein is expressed in 80% of ECs [41]. ERα-binding sites are highly conserved between tamoxifen-associated EC and BC but differ in non-tamoxifen-induced EC [41]. Phosphorylated ERα was previously investigated as a marker of transcriptional activation in the EC and BC of TCGA data sets [41]. Transcriptomic pathway analysis suggested that non-classical Erα signaling may be more important in EC and that the estrogen-associated transcriptome is tissue specific. Hence, exploration of ERα accompanied by transcriptional signaling data may help in the identification of a more appropriate biomarker signature in EC. In the TCGA dataset, around 16% of ECs have an amplification encompassing or overlapping *ESR1*, which results in ligand-binding domain (LBD) truncation in approximately 20% of cases [42]. 

In our study, no ESR1 mutation was identified. ESR1 LBD mutations are found in 5.8% of primary ECs with endometrioid histology [15,43,44]. ESR1 LBD mutations are mainly located at residues D538 and Y537 in a region essential for ligand binding and interactions with coregulatory proteins. The presence of ESR1 mutations is associated with obesity-independent EC, and patients with ESR1 LBD mutations tend to have a worse prognosis than patients with wild-type ESR1 tumors [43]. Interestingly, D538G mutant ESR1 confers estrogen-independent activity while causing additional regulatory changes in EC cells that are distinct from breast cancer cells [45]. This is relevant because ECs that are unable to bind estradiol may show estrogen-independent growth, making downstream elements of the ER pathway, alone or in combination with conventional endocrine therapy, a potentially attractive therapeutic strategy.

Interest to establish genomic profiles of mEC. While EC is a highly curable malignancy when uterine-confined, the prognosis for recurrent or metastatic disease is poor [46]. As shown in breast cancers, there is evidence that genomic alterations are also acquired during the evolution of cancers from their early to late stages and that the genomic landscape of early cancers is not representative of that of lethal cancers [47,48]. To date, the number of studies reporting genomic data in EC metastases is still not sufficient to understand the molecular mechanisms driving them. Genomic profiling of advanced EC might thus identify new potential therapeutic targets and contribute to optimizing endocrine therapy. In our study, *TP53, PIK3CA, PTEN, ARID1A, PPP2R1A, FGFR2, ATRX*, and *JAK3* mutations were mainly found to be maintained in the metastatic counterparts. This suggests that such alterations might be drivers of the primary tumor and necessary during evolution in an HT-resistance context. Among the gene mutations acquired in the metastatic counterparts, the most frequently affected pathways (PIK3/AKT/MTOR, epigenetic, cell cycle, and WNT/β-catenin) might confer a selective advantage to cancer metastasis formation including hormone resistance. Alterations targeting TP53, MTOR, and KMT2D, could be involved in hormone resistance and ne potentially targeted by recent therapies [49,50].

Various therapeutic strategies were previously reported to improve endocrine therapy efficiency in EC. To increase the efficacy of HT in endometrial cancer, various therapeutic strategies have been followed based on biological knowledge of ECs. They target mechanisms associated with certain pathways (i.e., epigenetic modulation (HDACs, DNMT, histone methylation), PI3K/AKT/MTOR, cell cycle, androgens, insulin/metabolic factors, and the immune microenvironment) [34]. 

There are still no effective treatments for EC and no agents to enhance sensitivity to progestin therapy. Among the markers of poor response, survivin (also called BIRC5), NRF2 (also called NFE2L2), and AKR1C1 appeared to be involved in the same pathway supporting the resistance to progestogens [51,52,53,54]. They were proposed as potential targets for new therapies against progestogen-resistant cases.

Recently LASS2 (also called CERS2, ceramide synthase 2) has been identified as a novel NRF2 target gene. In type I ECs, a feedback loop involves estrogen and the high expression of NRF2, which results in a low response to progestin and ultimately progestin resistance [55]. This provides in vitro proof-of-concept evidence that targeting NRF2/LASS2 may be a promising approach to inhibiting EC development and improving the efficacy of progestin therapy. Thus, as predictive biomarkers in EC, evaluating the overexpression of NRF2/LASS2 may also be useful to estimate cancer stage and potential resistance to progestin [55].

In patients with metastatic EC, MTOR inhibitors, such as temsirolimus, have been tested (in association or not with HT) [56]. However, temsirolimus efficacy and toxicity were disappointing, and the drug is not currently used in clinical practice. The MAPK pathway was also investigated due to its role in HR expression. Although promising results were obtained in vitro, these findings have not been tested in pre-clinical/clinical settings yet [57]. 

Therefore, without new treatments, better selection of the patients who might benefit from an old but effective, safe, and economic treatment, such as HT, represents an effective progress in EC management.

Better stratify patients with advanced EC. As we previously showed in advanced BC and other solid cancers, it is important in the future to systematically biopsy EC metastases to identify actionable genetic alterations (AGAs) and feed the literature to improve the knowledge on the molecular mechanisms driving them [47,58].

Because molecular alterations identify potential targeted therapies, to better stratify women with EC who will derive clinical benefit from new therapeutic options or who will exhibit resistance, it is crucial to develop precision medicine using extensive high-throughput molecular profiling. Additional predictive biomarkers/molecular signatures, and a better understanding of the ER/PR pathway biology should improve the responses to established endocrine therapies or their optimization, and favor the design of new therapeutic approaches. Additionally, in this stratification, the identification of patients with actionable genetic alterations (AGAs) that could be candidates for targeted therapies currently in development is paramount (e.g., prexasertib for EC harboring TP53 mutations; targeting the PI3K/AKT/MTOR pathways for EC harboring, for instance, PTEN and PIK3CA mutations; PARP inhibitors for homologous recombination-deficient EC; immune checkpoint inhibitors for EC with MSI; HER2 targeting EC harboring ERBB2 amplification/overexpression; EZH2 inhibitor or PARP inhibitors for EC harboring ARID1A mutations) [11,16,49,59]. Several clinical trials using HT alone or in combination with other drugs in advanced EC are already opened. Among them, NCT03643510, in which cancer response is evaluated in a prospective, single arm phase II study of the combination of CDK4 and CDK6 dual inhibitor abemaciclib and selective estrogen receptor degrader fulvestrant in hormone receptor positive recurrent endometrial carcinomas. Likewise, NCT04188548, a Phase 1a/1b study in which LY3484356, an oral selective estrogen receptor degrader (SERD), is administered as a monotherapy and in combination with anticancer therapies (aromatase inhibitor, abemaciclib, alpelisib, everolimus, trastuzumab, and pertuzumab) for patients with ER positive locally advanced or metastatic breast cancer or endometrial cancer (EMBER, phase 1a/1b).

Moreover, although *ESR1* alterations have a low penetrance in primary EC, this incidence may increase during disease evolution due to clonal selection by treatment pressure generating advanced EC/endocrine resistant tumors. Thus, the determination of *ESR1* mutation/amplification status in primary EC and in advanced EC with a non-invasive approach by using genomic profiling (at best on circulating DNA) may be relevant in the context of the response to endocrine therapy as well as in the follow-up of residual disease.

CBHT and LRHT. In our study, we reported 22 (57.9%) and 6 (15.8%) patients with CBHT and LRHT, respectively. While univariate analyses showed that PTEN mutations exhibited a trend for association with CBHT and an association with LRHT, neither oncogenic pathway alterations, including mutations and CNA, nor all variables tested were associated with CBHT and LRHT in multivariate analysis. 

Multiple small studies have demonstrated the clinical benefit of progestins, but variable response rates have been documented depending on the route of administration (IM versus oral), tumor grade, histology, PR expression status, and the line of therapy (i.e., first or subsequent exposure to endocrine therapy) [34]. Unfortunately, progestins have proven disappointing when evaluated in unselected EC populations in the adjuvant setting, with no clear benefit emerging for their use [60]. A variety of treatment approaches with progestins, selective ER modulators (SERMs), and aromatase inhibitors are available. The clinical efficacy of endocrine therapy and its optimization in EC was recently reviewed [34].

In our study, only patient’s age, mitotic index, and the presence of at least one HR (ER or PR) were considered to be prognostic factors for OS by multivariate analysis, while *PTEN* mutation was recently reported to be correlated with favorable prognosis in EC patients and found to be associated with immune infiltrating cells, such as Tregs and M1 macrophages, in the tumor microenvironment [61].

Study limitations. First, the retrospective nature of the study implies a selection bias based on a first selection of patients by clinicians for HT and a second selection based on the availability and exploitability of samples for molecular analysis. Second, the number of patients was low, although comparable with previous studies on HT for EC. Clearly, multicentric studies gathering more annotated samples and public data sharing are crucial to allow meta-analyses that will enhance our current knowledge of mEC genomics.

## 5. Conclusions

In conclusion, in this retrospective study, clinical and biological variables were simultaneously assessed to identify predictive factors of the response to HT in patients with metastatic EC. Unfortunately, no predictive factor was identified to stratify EC patients who will present CBHT and/or LRHT. Only patient’s age, mitotic index, and the presence of at least one HR (ER or PR) were considered to be prognostic factors for OS. The identification of gene mutations newly acquired in metastasis might help researchers to understand the formation of EC metastasis and define a personalized treatment for each patient with advanced EC.

## Figures and Tables

**Table 1:** Clinicopathological characteristics of patients with endometrial cancer.

Patient Characteristics		38 Patients Included (Interquantile Range or %)
Age in years old at diagnosis, median (IQR)		67 (61.25–74.50)
Weight in kg at diagnosis, median (IQR)		64 (56–75)
Patients with high blood pressure *n* = 33		18 (47.4%)
Patients with diabetes mellitus *n* = 32		5 (13.2%)
Surgical route of the primary EC		
	Laparoscopy	3 (7.9%)
	Open surgery	31 (81.6%)
	Robotic surgery	4 (10.5%)
FIGO stage of the primary EC		
	1A	7 (18.4%)
	1B	8 (21.1%)
	2	2 (5.3%)
	3A	7 (18.4%)
	3B	1 (2.6%)
	3C	8 (21.1%)
	4A	1 (2.6%)
	4B	4 (10.5%)
Pathological grade of the primary EC		
	1	11 (28.9%)
	2	17 (44.7%)
	3	10 (26.3%)
Pathological type (centralized review)		
	HGSEC	6 (15.8%)
	EEC	32 (84.2%)
Hormone therapy (metastatic EC)		
	Progestins	27 (71.1%)
	AI	7 (18.4%)
	Sequential AI—Progestins	2 (5.3%)
	SERM	2 (5.3%)
Number of treatments before HT in the metastatic setting		
	0	21 (54%)
	1	11 (28.9%)
	2	4 (10.5%)
	3	1 (2.6%)
	4	1 (2.6%)
First-line chemotherapy (metastatic EC) (*n* = 25)		
	Carboplatin–Paclitaxel	18 (72%)
	Carboplatin	3 (12%)
	Carboplatin + Other drug	1 (4%)
	Other drug	3 (12%)

Footnotes: FIGO: Fédération Internationale de Gynécologie et d’Obstétrique; EC: Endometrial Cancer; HGSEC: High Grade Serous Endometrial Cancer; EEC: Endometrioid Endometrial Cancer; AI: Aromatase Inhibitor; HT: hormone therapy.

**Table 2:** Results of univariate and multivariate analyses for CBHT.

Variables	Univariate		Multivariate	
	OR [95% CI]	*p*-Value	OR [95% CI]	*p*-Value
Age. *	0.97 [0.89–1.05]	4.63 × 10^−1^		
BMI > 30 (Yes vs. No)	1.18 [1.03–1.42]	**4.31 × 10^−2^**	Inf [0–Inf]	9.97 × 10^−1^
Mitotic index *	0.95 [0.90–0.99]	**4.49 × 10^−1^**	0.72 [0.20–1.02]	3.39 × 10^−1^
PR (Pos vs. Neg)	3.40 [0.86–14.69]	**8.62 × 10^−1^**		
RE (Pos vs. Neg)	2.67 [0.70–10.86]	1.57 × 10^−1^		
Hormone receptors (ER or PR) (Pos vs. Neg)	6.00 [1.15–46.35]	**4.74 × 10^−2^**	Inf [Inf–Inf]	9.97 × 10^−1^
Altered genome *	0.61 [0.37–0.89]	**3.33 × 10^−2^**	0.77 [NA–1.26]	4.65 × 10^−1^
MSI (MSS vs. MSI)	1.46 [0.24–9.02]	6.71 × 10^−1^		
Gene mutation (Mut vs. Wild)				
*PTEN*	5.20 [1.03–39.83]	**6.56 × 10^−2^**	Inf [0–NA]	9.97 × 10^−1^
*TP53*	0.44 [0.10–1.84]	2.65 × 10^−1^		
*PPP2R1A*	0.40 [0.07–2.00]	2.73 × 10^−1^		
*ARID1A*	1.14 [0.21–6.79]	8.76 × 10^−1^		
*FGFR2*	5.38 [0.74–110.60]	1.47 × 10^−1^		
*PIK3CA*	1.30 [0.19–11.05]	7.91 × 10^−1^		
*BCOR*	2.80 [0.32–60.38]	3.96 × 10^−1^		
*CTNNB1*	0.24 [0.01–2.09]	2.34 × 10^−1^		
*KMT2D*	0.81 [0.09–7.54]	8.46 × 10^−1^		
*NOTCH3*	Inf [0–Inf]	9.93 × 10^−1^		
*USH2A*	0.81 [0.09–7.54]	8.46 × 10^−1^		
*AKT1*	0 [NA–Inf]	9.94 × 10^−1^		
*ANK3*	0.38 [0.02–4.42]	4.52 × 10^−1^		
*APC*	1.75 [0.15–40.07]	6.62 × 10^−1^		
*ATRX*	1.75 [0.15–40.07]	6.62 × 10^−1^		
*CUX1*	1.75 [0.15–40.07]	6.62 × 10^−1^		
*EP300*	1.75 [0.15–40.07]	6.62 × 10^−1^		
*JAK3*	Inf [0–Inf]	9.94 × 10^−1^		
Oncogenic pathways alterations (Alt vs. non Alt)				
Cell Cycle	2.00 [0.33–16.30]	4.66 × 10^−1^		
HIPPO	0.20 [0.03–1.08]	**7.99 × 10^−2^**	12.23 [0–Inf]	7.33 × 10^−1^
MYC	0.87 [0.09–8.07]	8.94 × 10^−1^		
NOTCH	3.93 [0.92–19.32]	**7.33 × 10^−2^**	19.10 [0.14–3.9E06]	3.96 × 10^−1^
PI3K	0.71 [0.16–3.01]	6.48 × 10^−1^		
RTK_RAS	1.18 [0.23–6.11]	8.38 × 10^−1^		
TP53	0.48 [0.11–1.95]	3.08 × 10^−1^		
WNT	1.50 [0.33–7.31]	6.00 × 10^−1^		
BER	2.00 [0.33–16.30]	4.66 × 10^−1^		
NER	0.53 [0.06–3.72]	5.26 × 10^−1^		
FA	0.62 [0.12–2.91]	5.40 × 10^−1^		
HRD	0.46 [0.09–2.08]	3.20 × 10^−1^		
Other	0.52 [0.11–2.27]	3.91 × 10^−1^		

Footnotes: * Age, mitotic index, and altered genome are continuous variables. BMI: body mass index; PR: progesterone receptor; ER: estrogen receptor. All variables with *p* < 0.1 were in bold.

**Table 3:** Results of univariate and multivariate analysis for LRHT.

Variables	Univariate		Multivariate	
	OR [95% CI]	*p*-Value	OR [95% CI]	*p*-Value
Age. *	0.92 [0.79–1.03]	1.62 × 10^−1^		
BMI > 30 (Yes vs. No)	0.51 [0.02–4.01]	5.74 × 10^−1^		
Mitotic index *	0.99 [0.92–1.04]	6.43 × 10^−1^		
PR (Pos vs. Neg)	Inf [0.00–NA]	9.95 × 10^−1^		
RE (Pos vs. Neg)	1.20 [0.20–9.61]	8.46 × 10^−1^		
Hormone receptors (ER or PR) (Pos vs. Neg)	Inf [0.00–NA]	9.94 × 10^−1^		
Altered genome *	0.62 [0.19–1.03]	2.52 × 10^−1^		
MSI (MSS vs. MSI)	Inf [0.00–NA]	9.95 × 10^−1^		
Gene mutation (Mut vs. Wild)				
*PTEN*	7.00 [1.10–60.51]	**4.75 × 10^−2^**		
*TP53*	2.00 [0.31–12.85]	4.48 × 10^−1^		
*PPP2R1A*	1.75 [0.21–11.57]	5.69 × 10^−1^		
*ARID1A*	0.00 [NA–Inf]	9.94 × 10^−1^		
*FGFR2*	0.88 [0.04–7.32]	9.15 × 10^−1^		
*PIK3CA*	1.15 [0.05–10.20]	9.09 × 10^−1^		
*BCOR*	0.00 [NA–Inf]	9.96 × 10^−1^		
*CTNNB1*	0.00 [NA–Inf]	9.96 × 10^−1^		
*KMT2D*	1.60 [0.07–15.90]	7.08 × 10^−1^		
*NOTCH3*	1.60 [0.07–15.90]	7.08 × 10^−1^		
*USH2A*	1.60 [0.07–15.90]	7.08 × 10^−1^		
*AKT1*	0.00 [NA–Inf]	9.94 × 10^−1^		
*ANK3*	0.00 [NA–Inf]	9.94 × 10^−1^		
*APC*	2.50 [0.10–31.67]	4.87 × 10^−1^		
*ATRX*	0.00 [NA–Inf]	9.94 × 10^−1^		
*CUX1*	2.50 [0.10–31.67]	4.87 × 10^−1^		
*EP300*	0.00 [NA–Inf]	9.94 × 10^−1^		
*JAK3*	2.50 [0.10–31.67]	4.87 × 10^−1^		
Oncogenic pathways alterations (Alt vs. non Alt)				
Cell Cycle	0.00 [NA–Inf]	9.95 × 10^−1^		
HIPPO	0.71 [0.03–5.95]	7.79 × 10^−1^		
MYC	0.00 [NA–Inf]	9.96 × 10^−1^		
NOTCH	2.18 [0.31–18.73]	4.32 × 10^−1^		
PI3K	Inf [0.00–NA]	9.95 × 10^−1^		
RTK_RAS	0.43 [0.06–3.82]	4.08 × 10^−1^		
TP53	2.18 [0.31–18.73]	4.32 × 10^−1^		
WNT	0.50 [0.02–4.04]	5.60 × 10^−1^		
BER	1.10 [0.05–9.77]	9.38 × 10^−1^		
NER	1.44 [0.06–13.57]	7.70 × 10^−1^		
FA	0.59 [0.03–4.86]	6.63 × 10^−1^		
HRD	0.50 [0.02–4.04]	5.60 × 10^−1^		
Other	2.35 [0.29–49.50]	4.71 × 10^−1^		

Footnotes: *Age, mitotic index, and altered genome are continuous variables. BMI: body mass index; PR: progesterone receptor; ER: estrogen receptor. All variables with *p* < 0.1 were in bold.

**Table 4:** Results of univariate and multivariate analyses for OS.

Variables	Univariate		Multivariate	
	OR [95% CI]	*p*-Value	OR [95% CI]	*p*-Value
Age. *	1.09 (1.03–1.15)	**1.50 × 10^−3^**	1.09 [1.02–1.15]	**8.47** **× 10^−3^**
BMI > 30 (Yes vs. No)	0.86 (0.34–2.17)	7.50 × 10^−1^		
Mitotic index *	1.04 (1.01–1.06)	**5.80 × 10^−3^**	1.04 [1.00–1.08]	**3.73** **× 10^−2^**
PR (Pos vs. Neg)	0.38 (0.18–0.80)	**1.00 × 10^−2^**		
RE (Pos vs. Neg)	0.79 (0.39–1.63)	5.30 × 10^−1^		
Hormone receptors (ER or PR) (Pos vs. Neg)	0.29 (0.13–0.67)	**3.60 × 10^−3^**	0.21 [0.07–0.58]	**2.67** **× 10^−3^**
Altered genome *	1.07 (1.01–1.14)	**2.90 × 10^−2^**	0.98 [0.90–1.07]	6.42 × 10^−1^
MSI (MSS vs. MSI)	0.53 (0.21–1.34)	1.80 × 10^−1^		
Gene mutation (Mut vs. Wild)				
*PTEN*	0.70 (0.30–1.60)	3.90 × 10^−1^		
*TP53*	1.08 (0.49–2.39)	8.40 × 10^−1^		
*PPP2R1A*	0.83 (0.33–2.10)	7.00 × 10^−1^		
*ARID1A*	2.16 (0.86–5.43)	1.00 × 10^−1^		
*FGFR2*	1.19 (0.45–3.16)	7.30 × 10^−1^		
*PIK3CA*	0.91 (0.31–2.67)	8.70 × 10^−1^		
*BCOR*	1.30 (0.44–3.82)	6.40 × 10^−1^		
*CTNNB1*	1.17 (0.40–3.44)	7.70 × 10^−1^		
*KMT2D*	0.59 (0.14–2.49)	4.70 × 10^−1^		
*NOTCH3*	0.78 (0.23–2.61)	6.80 × 10^−1^		
*USH2A*	0.73 (0.22–2.45)	6.10 × 10^−1^		
*AKT1*	1.21 (0.36–4.04)	7.60 × 10^−1^		
*ANK3*	1.15 (0.27–4.97)	8.50 × 10^−1^		
*APC*	1.40 (0.42–4.72)	5.80 × 10^−1^		
*ATRX*	1.50 (0.44–5.19)	5.20 × 10^−1^		
*CUX1*	0.72 (0.17–3.06)	6.60 × 10^−1^		
*EP300*	2.12 (0.60–7.49)	2.40 × 10^−1^		
*JAK3*	0.59 (0.14–2.53)	4.80 × 10^−1^		
Oncogenic pathways alterations (Alt vs. non Alt)				
Cell Cycle	1.33 (0.44–4.00)	6.10 × 10^−1^		
HIPPO	1.29 (0.51–3.25)	5.90 × 10^−1^		
MYC	2.10 (0.71–6.23)	1.80 × 10^−1^		
NOTCH	0.72 (0.32–1.59)	4.20 × 10^−1^		
PI3K	0.63 (0.28–1.42)	2.60 × 10^−1^		
RTK_RAS	1.73 (0.68–4.42)	2.50 × 10^−1^		
TP53	1.05 (0.48–2.31)	9.00 × 10^−1^		
WNT	1.11 (0.49–2.50)	8.00 × 10^−1^		
BER	0.97 (0.36–2.60)	9.60 × 10^−1^		
NER	1.23 (0.42–3.60)	7.10 × 10^−1^		
FA	1.37 (0.60–3.17)	4.60 × 10^−1^		
HRD	1.07 (0.46–2.46)	8.80 × 10^−1^		
Other	1.51 (0.65–3.49)	3.40 × 10^−1^		

Footnotes: * Age, mitotic index, and altered genome are continuous variables. BMI: body mass index; PR: progesterone receptor; ER: estrogen receptor. All variables with *p* < 0.1 were in bold.

## Data Availability

aCGH data have been deposited in the ArrayExpress database at EMBL-EBI (www.ebi.ac.uk/arrayexpress, accessed on 8 March 2022) under accession number E-MTAB-11552.

## Supplementary Materials

The following supporting information can be downloaded at: https://www.mdpi.com/article/10.3390/jpm12050655/s1, Table S1: Antibodies used for immunohistochemistry analyses; Table S2: Gene panel “TSV9 FFPE” (Agilent Design ID: 27066-1418742171) used in tNGS to identify mutation in 495 genes; Table S3: Genes involved in oncogenic pathways; Table S4: List of mutated genes; Table S5: Gistic analysis: amplified regions; Table S6: Gistic analysis: deleted regions; Table S7: Gistic analysis: amplified driver genes defined by CGI; Table S8: Oncogenic pathways altered in EC primary tumors and metastases samples; Table S9: Oncogenic pathways altered in the seven primary EC tumors and metastases pairs; Table S10: Comparison between gene mutations observed in our cohort and 103 mEC samples from MSKCC [36]; Table S11: Comparison between gene CNA observed in our cohort and in 103 mEC samples from MSKCC [36]. Figure S1: Kaplan–Meier overall survival of 38 patients with endometrial carcinoma.
