# Supplementary material for: Investigation of Molecular Features Involved in Clinical Responses and Survival in Advanced Endometrial Carcinoma Treated by Hormone Therapy
Source: J Pers Med. 2022 Apr 19;12(5):655. doi: 10.3390/jpm12050655 (PMC9143816; doi:10.3390/jpm12050655)
Supplement: Supplementary file 1 [file jpm-12-00655-s001.zip › Supplementary Figure Legends.pdf]

**Supplementary Figure Legends**

**Supplementary Figure S1.** Kaplan-Meier overall survival of 38 patients with endometrial carcinoma. Censored values (+) indicate the last known follow-up time for those subjects still alive. Confidence interval are depicted by the grey color.
