# Supplementary figures and images for: Investigation of Molecular Features Involved in Clinical Responses and Survival in Advanced Endometrial Carcinoma Treated by Hormone Therapy
Source: J Pers Med. 2022 Apr 19;12(5):655. doi: 10.3390/jpm12050655 (PMC9143816; doi:10.3390/jpm12050655)

**Fig. S1**

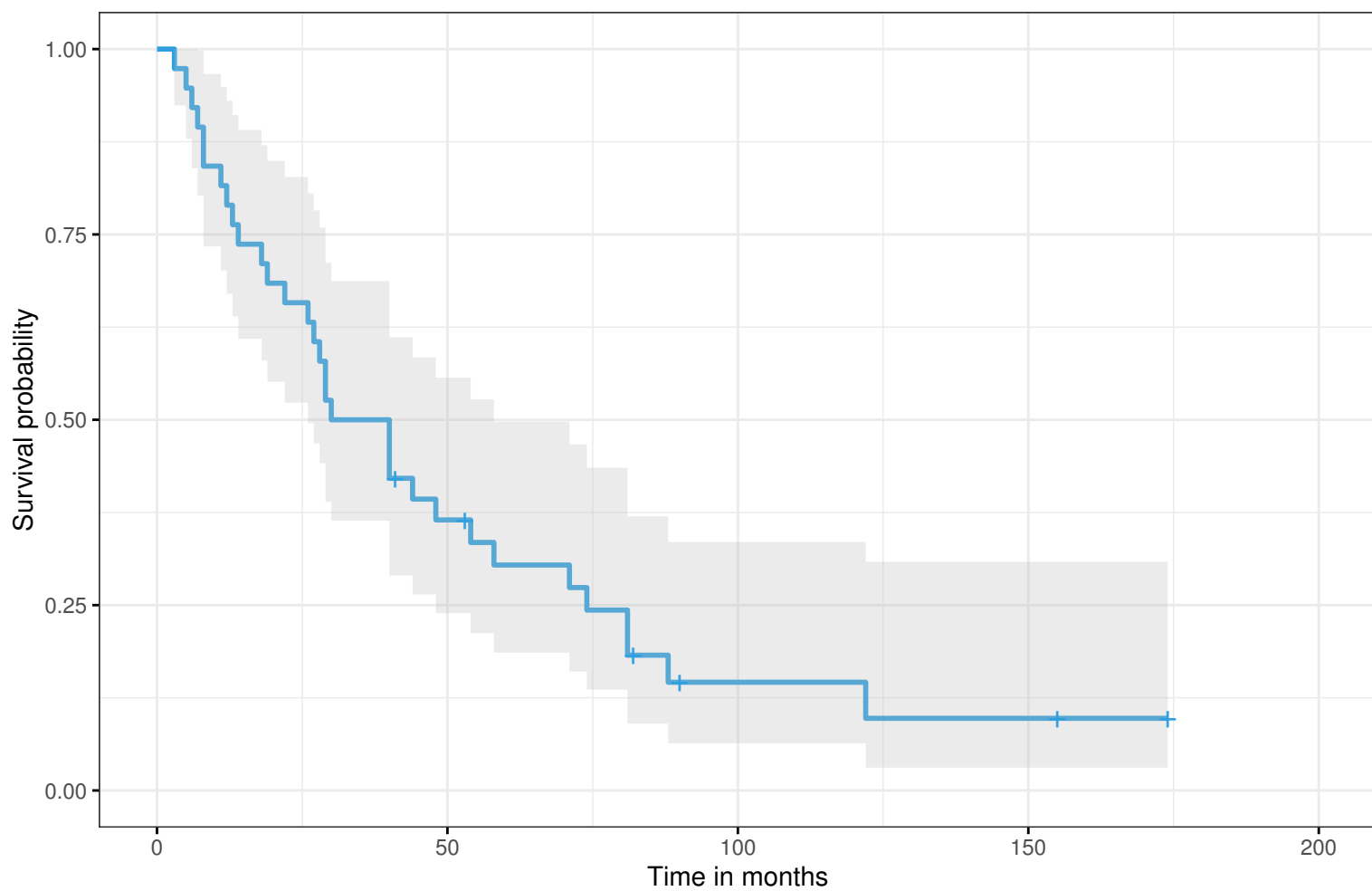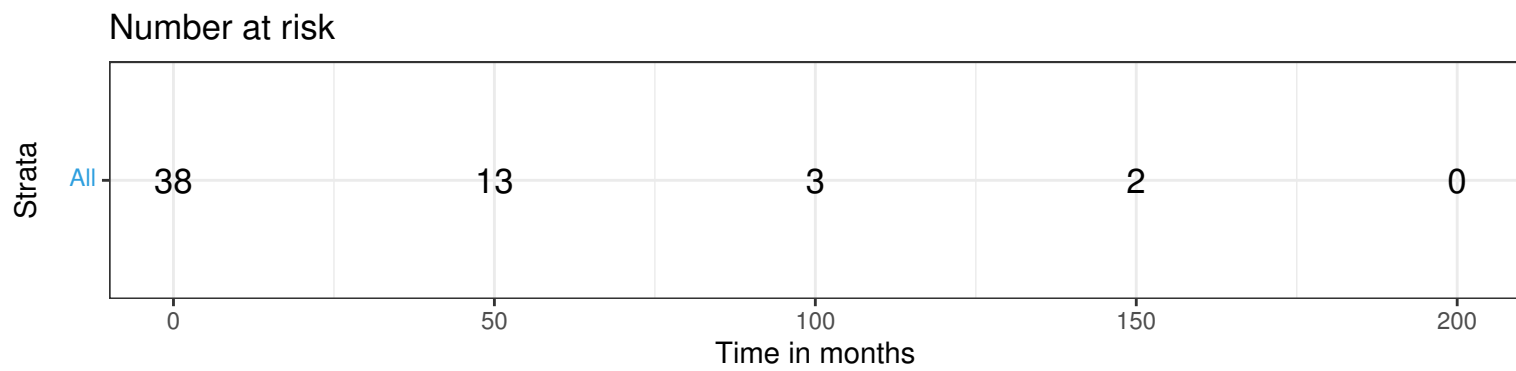

Supplement: Supplementary file 1 [file jpm-12-00655-s001.zip › Figure_S1.pdf]
